# CCL18 from ascites promotes ovarian cancer cell migration through proline-rich tyrosine kinase 2 signaling

**DOI:** 10.1186/s12943-016-0542-2

**Published:** 2016-09-09

**Authors:** Denis Lane, Isabelle Matte, Claude Laplante, Perrine Garde-Granger, Alex Carignan, Paul Bessette, Claudine Rancourt, Alain Piché

**Affiliations:** 1 Département de Microbiologie et Infectiologie, Université de Sherbrooke, 3001, 12ième Avenue Nord, Sherbrooke, Québec J1H 5N4 Canada; 2 Département de Pathologie, Université de Sherbrooke, 3001, 12ième Avenue Nord, Sherbrooke, J1H 5N4 Canada; 3 Service d’obstétrique et gynécologie, Département de Chirurgie, Faculté de Médecine, Université de Sherbrooke, 3001, 12ième Avenue Nord, Sherbrooke, J1H 5N4 Canada

**Keywords:** Pyk2, Ovarian carcinoma, Ascites, CCL18, Cell migration

## Abstract

**Background:**

Ovarian cancer (OC) ascites consist in a proinflammatory tumor environment that is characterized by the presence of various cytokines, chemokines and growth factors. The presence of these inflammatory-related factors in ascites is associated with a more aggressive tumor phenotype. CCL18 is a member of CCL chemokines and its expression has been associated with poor prognosis in some cancers. However, its role in OC progression has not been established. Therefore, the aim of the current study was to elucidate the role of ascites CCL18 in OC progression.

**Methods:**

ELISA and tissue microarrays were used to assess CCL18 in ascites and phospho-Pyk2 expression in cancer tissues respectively. Cell migration was assessed using Boyden chambers. CCL18 and ascites signaling was examined in ovarian cancer cells utilizing siRNA and exogenous gene expression.

**Results:**

Here, we show that CCL18 levels are markedly increased in advanced serous OC ascites relative to peritoneal effusions from women with benign conditions. Ascites and CCL18 dose-dependently enhanced the migration of OC cell lines CaOV3 and OVCAR3. CCL18 levels in ascites positively correlated with the ability of ascites to promote cell migration. CCL18 blocking antibodies significantly attenuated ascites-induced cell migration. Ascites and CCL18 stimulated the phosphorylation of proline-rich tyrosine kinase 2 (Pyk2) in CaOV3 and OVCAR3 cells. Most importantly, the expression of phosphorylated Pyk2 in serous OC tumors was associated with shorter progression-free survival. Furthermore, enforced expression of Pyk2 promoted tumor cell migration while siRNA-mediated downregulation of Pyk2 attenuated cell migration. Downregulation of Pyk2 markedly inhibited ascites and CCL18-induced cell migration.

**Conclusions:**

Taken together, our findings establish an important role for CCL18, as a component of ascites, in the migration of tumor cells and identify Pyk2 as prognostic factor and a critical downstream signaling pathway for ascites-induced OC cell migration.

**Electronic supplementary material:**

The online version of this article (doi:10.1186/s12943-016-0542-2) contains supplementary material, which is available to authorized users.

## Background

Ovarian cancer (OC) is the second most frequent gynecological cancer and is associated with a poor prognosis because OC progression is often asymptomatic and is detected at a late stage. Widespread intraperitoneal metastasis is the major cause of mortality among OC patients [1, 2]. It results from a sequential process in which tumor cells shed from the primary tumor into ascites are disseminated throughout the peritoneal cavity [1, 2]. Most OC patients present with advanced diseases (stage III/IV) with large amount of ascites. The presence of ascites at diagnostic correlates with peritoneal spread of the tumor and with a decreased 5-year survival rate [3–5]. A variety of cytokines, chemokines and growth factors are present in OC ascites [6–8]. There is growing evidence that inflammatory cytokines/chemokines within ascites lead to a state of chronic inflammation [9, 10]. Chronic inflammation, in turn, contributes to tumor progression by creating a proliferative, migrating and prosurvival environment [11, 12]. Indeed, OC ascites have been shown to enhance tumor cell proliferation, migration and survival [13–17].

Chemokines are important components of cancer-related inflammation and, in the tumor environment, may play a pivotal role in tumor progression and metastasis. Chemokine (C-C motif) ligand 18 (CCL18), which is predominately produced by tumor-associated macrophages (TAMs), promote the migration and invasion of breast cancer cells [18, 19]. CCL18 also correlates with metastasis and poor prognosis of patients with breast cancer [20]. CCL18 is expressed at higher levels in OC ascites compared to none ovarian carcinomas [21]. Its tissue expression correlates with metastasis in OC patients [22]. Serum levels of CCL18 has been examined as a potential biomarker for discriminating patients with OC versus those with benign gynecological conditions [23].

CCL18 effects on migration and invasion of breast cancer cells are mediated by the proline-rich tyrosine kinase 2 (Pyk2) [19]. Pyk2 is a cytoplasmic tyrosine kinase that belongs to the focal adhesion kinase (FAK) family [24]. CCL18 specifically binds PITPNM3/Nir1 receptor and activates Pyk2 by phosphorylation of tyrosine residue Y402 that functions as a docking site for the SH2 domain of Src [25]. Pyk2 regulates different signal transduction cascade that control cell proliferation, migration and invasion [20, 22, 26, 27]. Pyk2 is overexpressed in hepatocellular carcinoma (HCC) cells and its expression is associated with poor prognosis [25]. Enforced expression of Pyk2 promotes migration and invasion in HCC cells via the activation of ERK pathway [27, 28]. Little is known however about the role Pyk2 in OC cells.

In this study, we aim to investigate the contribution of the tumor environment to CCL18/Pyk2 signaling and the migration of OC cells. We demonstrate that high levels of CCL18 are present in OC ascites and that CCL18 is an important component of ascites for the ascites-mediated migration of OC cells. Ascites and CCL18 stimulate the phosphorylation and expression of Pyk2, which is critical for mediated CCL18-induced migration.

## Methods

### Patients

Ascites are routinely obtained at the time of the debulking surgery of ovarian cancer patients treated at the Centre Hospitalier Universitaire de Sherbrooke. After collection, cell-free ascites are stored at −80 °C in our tumor bank until use. The study population consisted of 53 women with newly diagnosed epithelial ovarian cancer admitted at the Centre Hospitalier Universitaire de Sherbrooke. Twenty four cases with histologically benign gynecological conditions including fibromas, endometriosis, and mucinous and serous cystadenomas constituted the control group. This study was approved by the Institutional Review Board of the Centre de Recherche Étienne-Le Bel. Informed consent was obtained from women that underwent surgery by the gynecologic oncology service between 2000 and 2014. All samples were reviewed by an experienced pathologist. Baseline characteristics and serum CA125 levels were collected for all patients. All patients had a follow up ≥12 months. Disease progression was defined by either serum CA125 ≥2 X nadir value on two occasions, documentation of lesion progression or appearance of new lesions on CT-scan or death. Patient’s conditions were staged according to the criteria of the International Federation of Gynecology and Obstetrics (FIGO). PFS was defined by the time from the initial surgery to evidence of disease progression.

### Cell culture and reagents

The human OC cell lines CaOV3 and OVCAR3 were obtained from the American Type Culture Collection (Manassas, VA) and maintained in a humidified 5 % CO_2_ incubator at 37 °C. Cells were passaged twice weekly. OVCAR3 cells were maintained in RPMI-1640 (Wisent, St-Bruno, QC, Canada) supplemented with 20 % FBS, insulin (10 mg/L), glutamine (2 mM) and antibiotics. CaOV3 cells were cultured in DMEM/F12 (Wisent) supplemented with 10 % FBS, 2 mM glutamine and antibiotics. Acellular ascites fractions were obtained at the time of initial cytoreductive surgery from women with advanced serous ovarian carcinomas. Samples were supplied by the Banque de tissus et de données cliniques et biologiques sur les cancers gynécologiques et du sein de Sherbrooke as part of the Banque de tissus et de données du Réseau de Recherche en Cancer des Fonds de Recherche du Québec en Santé (FRQS) affiliated to the Canadian Tumor Repository Network (CTRNet). HRP-conjugated anti-mouse and rabbit antibodies and anti-FAK antibody were purchased from Cell Signaling Technology (Danvers, MA). Anti-phospho FAK and anti-phospho Pyl2 were from Thermo Fisher (Waltham, MA). Anti-Pyk2 and anti-Tubulin were purchased from Sigma-Aldrich (Oakville, ON). CCL18 and CCL18 neutralizing antibody were from RnD Systems (Minneapolis, MN). Plasmid pCMV6-ENTRY-PTK2B was obtained from Origene (Rockville, MD).

### Quantitative real time PCR

Total RNA was extracted from CaOV3 cells using TRIzol reagent (Life Technologies) according to the manufacturer’s protocol and subjected to reverse transcription (RT) with oligodT from Promega (Madison, WI) and MMULV reverse transcriptase enzyme. RNA concentrations were quantified by measurement of absorbance at 260 nm. The integrity of the cDNA was assessed with the Taqman gene expression assays (Life Technologies), done on *RPLPO* housekeeping gene. Each sample was normalized to the housekeeping gene levels. Primers for Pyk2 are as follow: Forward: 5′-CGGACTGATGACCTGGTGTA-3′, Reversed: 5′-TTCTTCACCACCACCACGTA-3′. Cycle conditions for all PCRs were as follow: an initial incubation of 2 min at 95 °C followed by 35 cycles at 94 °C 30 s, 55 °C 30 s, 72 °C 60 s. The 2^-ΔΔCt^ method was used to calculate the relative levels of specific mRNA.

### Migration assay

Cells (5 × 10^3^) were suspended in 500 μl FBS and hormone-free DMEM/F12 and were seeded in the top chamber of monolayer-coated polyethylene terephthalate membranes cell culture inserts (24-wells insert, 8 μm pore size). The bottom chamber contained 0.75 ml DMEM/F12 supplemented with 10 % fetal bovine serum, 10 % ascites, or CCL18. The cells were incubated for 16–20 h, and cells that did not migrate through the membrane were removed by scraping with a cotton swab. Cells that migrated through the membrane were fixed with ice cold methanol for 10 min and stained with a 0.5 % crystal violet, 20 % (*v/v*) hematoxylin solution in ethanol for 15 min. After several washes in PBS, membranes were allowed to dry before being mounted on a glass slide. Ten random fields were counted at × 100 magnification.

### ELISA measurements

Cytokine levels in peritoneal fluid samples were determined by ELISA using the commercially available human Quantikine kits from R&D Systems (Minneapolis, MN). The assays were performed in duplicate according to the manufacturer’s protocols. The detection threshold was 1.1 ng/ml for CCL18. The intra-assay variability was 3.2–3.7 % for CCL18. The inter-assay variability was 3.5 %. All samples were examined in duplicate and the median values were used for statistical analysis.

### Western blot analysis

Cells were harvested and washed with ice-cold PBS. Whole cell extracts were prepared in lysing buffer (glycerol 10 %, Triton X-100 1 %, TRIS 10 mM pH 7.4, NaCl 100 mM, EGTA 1 mM, EDTA 1 mM, SDS 0.1 %) containing protease inhibitors (0.1 mM AEBSF, 5 μg/ml pepstatin, 0.5 μg/ml leupeptin and 2 μg/ml aprotinin) and phosphatase inhibitors (Na_4_P_2_O_7_ 20 mM, NaF 1 mM, Na_3_VO_4_ 2 mM). Proteins were separated by 12 % SDS-PAGE gels. Proteins were transferred to PVDF membranes (Roche, Laval, Québec, Canada) by electroblotting, and immunoblot analysis was performed as previously described [13]. All primary antibodies were incubated overnight at 4 °C in 5 % fat-free milk. Proteins were visualized by enhanced chemiluminescence (GE Healthcare, Baie d’Urfé, Québec, Canada).

### siRNA transfections

The Fluorescein-labeled Luciferase GL2 duplex or a non-target (scrambled) siRNAs used as a control were from Dharmacon Research (Lafayette, CO). Cells (6 × 10^4^) were seeded in 6-well plates and allowed to adhere for 24 h. Cells (50 % confluent) were transfected with a mixture containing Lipofectamine 2000™ (Life Technologies), optiMEM (Life Technologies) and siRNA (10 μM). The siRNAs/Lipofectamine complex was then added to the media of 6-well plates containing cells. Cells were incubated for 4–6 h at 37 °C in a CO_2_ incubator and medium containing FBS was then added. The Pyk2 smart pool siRNAs was from GE Health Care (Ottawa, Canada).

### Immunohistochemistry staining

TMAs were acquired from the Pan-canadian platform for the development of biomarker-driven subtype specific management of ovarian carcinoma (COEUR study). Slides were deparaffinized in citrate buffer containing 0.05 % Tween at 97 °C for 20 min, washed with PBS and incubated with 3 % peroxide. After treatment, slides were submerged in a citrate buffer (0.01 M citric acid, pH 6.0) for 15 min, and incubated with a protein blocking serum-free reagent (Dako Canada). The TMAs were stained by an immunoperoxidase method using an automated tissue immunostainer (Dako Canada) with DABchromogen. The TMAs were counter stained with hematoxilin and slides were scanned using HAMAMATSU Nanozoomer (Boston, MA) and each tissue section were scored according to the staining intensity where negative staining = 0, low staining = 1+, moderate staining = 2+ and high staining = 3+. Two separate TMAs containing different section of the same tumor were scored and the mean intensity was used.

### Statistical analysis

Statistical comparisons between two groups were performed using the Mann-Whitney or Student’s *t*-test. The correlation between phospho-FAK and phospho-Pyk2 expression in tissue section was determined by the Pearson’s correlation test. Statistical differences in PFS or overall survival were determined by the log-rank test, and Kaplan-Meier survival curves were made. PFS was defined as the interval between the date of the initial debulking surgery and the time of disease progression or the last date of follow up. Statistical significance was indicated by *P* < 0.05.

## Results

### Levels of CCL18 in malignant ascites from ovarian cancer patients

**Fig. 1:**
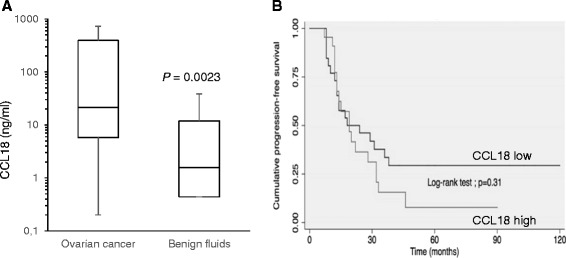
Presence of CCL18 in ovarian cancer ascites. **a** The concentration of CCL18 in advanced serous ovarian cancer ascites (*n* = 54) and benign fluids (*n* = 24) was determined by ELISA. Box plot representing levels of CCL18. **b** Kaplan-Meier curve of ascites CCL18 levels. The median CCL18 level (21.7 ng/ml) was taken as the cutoff point

A previous study has shown that levels of CCL18 in ascites from patients with OC were significantly higher compared to those with none ovarian carcinomas [21]. To extent these data, we measured the CCL18 levels in ascites of patients with serous OC (*n* = 53) and in the peritoneal fluids of patients with benign gynecological conditions (*n* = 24). CCL18 levels were significantly higher in OC ascites compared to benign fluids with median concentrations of 21.4 ng/ml and 1.5 ng/ml respectively (*P* = 0.0023) (Fig. 1). These data raise the possibility that CCL18 in ascites could play a role in OC progression.

We next correlated the levels of CCL18 in ascites with the progression-free survival (PFS). Women were separated in CCL18 high and low groups based on the CCL18 median level in ascites. A Kaplan-Meier progression-free survival curve showed that, although it did not reach statistical significance, women with low CCL18 (<21.4 ng/ml) had a tendency for longer PFS with a median of 21 months compared to 18 months for those with high CCL18 (>21.7 ng/ml) level in ascites (log rank test, *P* = 0.31) (Fig. 1b).

### Malignant ascites and CCL18 stimulate the migration of ovarian cancer cells

**Fig. 2:**
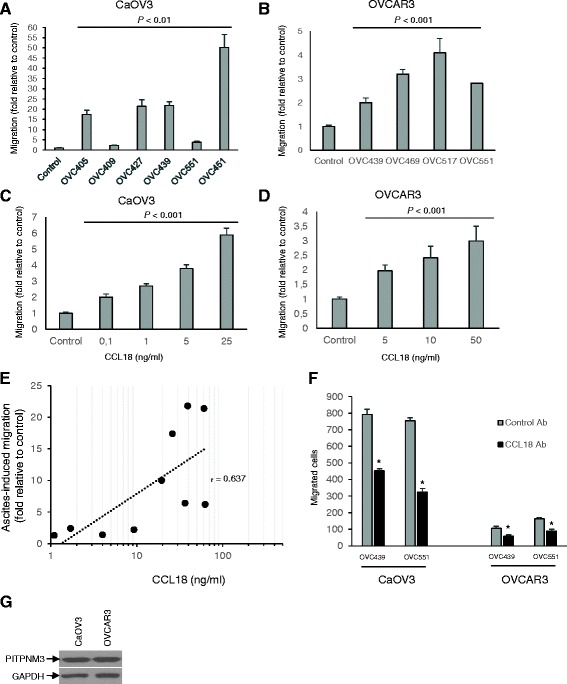
Ovarian cancer ascites and CCL18 enhance the migration of tumor cells. The migration of ovarian cancer CaOV3 cells (**a**) and OVCAR3 cells (**b**) was evaluated at 24 h using Boyden chambers with either serum-free medium (control) or different ovarian cancer ascites as chemoattractants. CaOV3 cells (**c**) and OVCAR3 cells (**d**) were treated with 0–50 ng/ml CCL18 for 24 h and migration was determined. Results shown are representative of three independent experiments and data are expressed as fold increased relative to control. *P* values are indicated relative to controls. **e** CCL18 levels in ascites were correlated with the ability of ascites to stimulate CaOV3 cell migration. The correlation coefficient (*r*) was determined by Pearson’s correlation coefficient test. **f** Incubation of ascites (OVC439 and OVC551) with CCL18-blocking antibody (20 μg/ml) reduces the migration expressed as the number of migrated cells in CaOV3 and OVCAR3 cells. Values as mean ± SEM and represent three independent experiments: *P < 0.001. **g** Immunoblot analysis of PITPNM3 expression in CaOV3 and OVCAR3 cells

We first examined the effect of serous OC ascites on the migration of OC cells CaOV3 and OVCAR3, two commonly used OC cell line models. CaOV3 cells were exposed to different ascites (10 % v/v) obtained from patients with advanced serous OC and migration was determined using Transwell chambers. The concentration of ascites used in this study was based on previous studies demonstrating a biological effect of ascites on OC cells at this concentration [13–15]. Although the magnitude of the effect on cell migration was variable, all 6 OC ascites samples significantly enhanced migration of CaOV3 cells (Fig. 2a). Migration was also enhanced by serous OC ascites when tested with OVCAR3 cells (Fig. 2b). Unlike CaOV3 cells, OVCAR3 cells are non-invasive in Boyden chambers and non-motile on plastic [29]. In line with the low motility potential of OVCAR3 cells, the magnitude of ascites-mediated migration was lower in these cells relative to CaOV3. We next found that CCL18 dose-dependently induced the migration of CaOV3 and OVCAR3 cells (Fig. 2c and d). The range of CCL18 concentrations selected were within the range of those found in OC ascites. Furthermore, there was a positive correlation between the levels of CCL18 found in serous OC ascites and the ability of these ascites to induce the migration of CaOV3 cells (*r* = 0.637, *P* < 0.01) (Fig. 2e). These data suggest that CCL18 and serous OC ascites enhance OC cell migration and that CCL18 may have a role in ascites-induced cell migration.

To further define the role of CCL18 in ascites-induced cell migration, we examined the effect of a CCL18-blocking antibody. As shown in Fig. 2f, the ascites-induced migration of OC cells was significantly inhibited by the preincubation of ascites with the CCL18-blocking antibody. Although significant, the CCL18-blocking antibody only achieved a partial (43 to 57 %) inhibition of ascites-induced migration suggesting the possible contribution of other factors in this process. Nonetheless, our data suggest that CCL18 is an important factor in ascites that promotes the migration of OC cells. The difference in the magnitude of ascites-induced effect on migration between CaOV3 and OVCAR3 cells was not related the CCL18 receptor expression as PITPNM3 levels were found to be similar in both cell lines (Fig. 2g).

### Ascites and CCL18 induce activation of Pyk2 in ovarian cancer cells

**Fig. 3:**
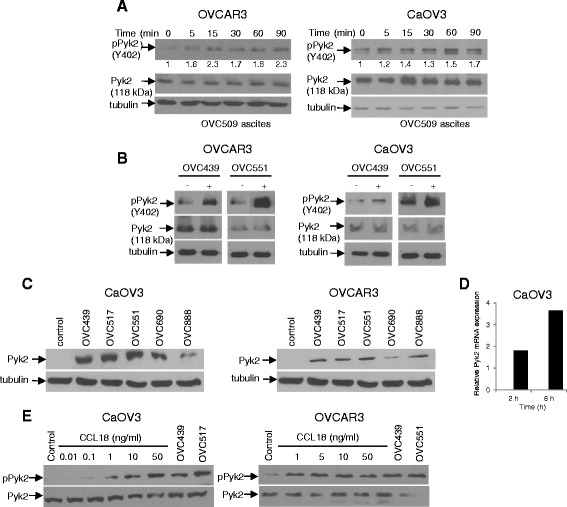
Ovarian cancer ascites and CCL18 induce the phosphorylation of Pyk2. **a** Serous ovarian cancer ascites OVC509 induce a rapid phosphorylation of Pyk2 at tyr402 in OVCAR3 and CaOV3 cells. Cells were incubated in serum-free media for 24 h, the media was change and OVC509 ascites (10 % *v/v*) was added for up to 90 min. Densitometric quantification of phosphorylated Pyk2 normalized to total Pyk2. The fold increase of phosphorylated Pyk2 is indicated at the bottom of pPyk2 panels. **b** The stimulation of Pyk2 phosphorylation was confirmed in CaOV3 and OVCAR3 cells using the additional serous ovarian cancer ascites, OVC439 and OVC551. **c** Immunoblot for total Pyk2 expression from CaOV3 and OVCAR3 lysates obtained at 24 h following the addition of ovarian cancer ascites. **d** Real-time PCR analysis of Pyk2 transcript levels at 2 h and 6 h following the addition of OVC439 ascites. Results were standardized using primers of the housekeeping gene RPLPO. Results are expressed as fold change relative to basal levels observed in cells incubated in the absence of ascites. **e** CaOV3 and OVCAR3 cells were treated with increasing concentration of CCL18 (0–50 ng/ml) or ascites for 90 min. Immunoblot were obtained to assess the phosphorylation of Pyk2

Previous studies have shown that CCL18 exerts its effect on cell migration through Pyk2 activation in breast cancer cells [19]. To investigate whether OC ascites affect Pyk2 activation or expression, immunoblots were performed at different time points in CaOV3 and OVCAR3 cells in the presence of OVC509 serous ascites. As shown in Fig. 3a, Pyk2 was rapidly activated in a time-dependent manner by the addition ascites to the culture medium of CaOV3 and OVCAR3 cells. The activation of Pyk2 by OC ascites in CaOV3 and OVCAR3 cells was confirmed using two other ascites samples, OVC439 and OVC551 (Fig. 3b). We next determine whether the expression of Pyk2 was affected by OC ascites. As shown in Fig. 3, the expression of Pyk2 protein was upregulated when CaOV3 and OVCAR3 cells were incubated with different serous OC ascites. To determine whether Pyk2 expression changes were the result of increased transcription or altered protein stability, we examined Pyk2 mRNA levels in CaOV3 cells at 2 and 6 h following exposure to OVC439 ascites. Pyk2 mRNA levels, as determined by quantitative real time PCR, were upregulated by at least two-fold (Fig. 3d). Treatment of CaOV3 and OVCAR3 cell lines with CCL18 (0–50 ng/ml) for 90 min induced the activation of Pyk2 in a dose-dependent manner to a level similar to that found with OC ascites (Fig. 3e). These data demonstrate that OC ascites and CCL18 induce Pyk2 activation in OC cells.

### Pyk2 activation in advanced serous ovarian cancer

**Fig. 4:**
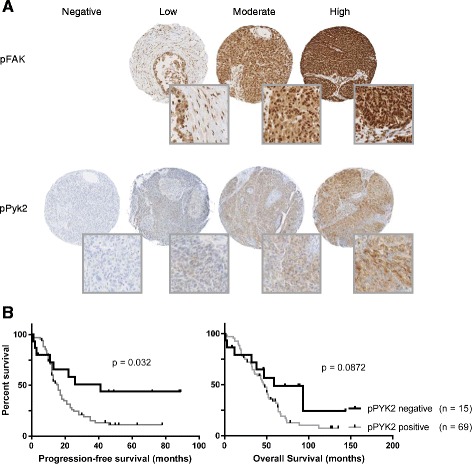
Activation of Pyk2 in advanced serous ovarian cancer. A tissue microarray containing sections of 120 tumors from women with advanced serous ovarian cancer was stained with antibodies specific for pPyk2 and pFAK. Tumors were scored as negative, weakly positive (1+), moderately positive (2+) or intensively positive (3+). **a** Representative images of immunoperoxidase-stained tissue for pFAK and pPyk2 demonstrating either negative staining (0), weak (1+), moderate (2+) and intensive staining (3+). None of the tumors stained negative for pFAK. **b** Kaplan-Meier curves for progression-free and overall survival. The graphs show women who had advanced serous ovarian carcinoma with pPyk2 positive tissue staining and women who had advanced serous ovarian carcinoma with no pPyk2 expression

Pyk2 is a non-receptor tyrosine kinase structurally related to FAK, which integrate growth factor and integrin signals to promote cell migration [30, 31]. Tissue microarrays from women with high grade advanced serous OC were immunohistochemically stained for pPyk2 and pFAK (Fig. 4a). Previous reports demonstrated that Pyk2 is localized in the cytoplasm and the perinuclear region [32, 33]. In stress-induced tumor cells, Pyk2 can accumulate in nucleus [32]. As shown in Fig. 4a, pPyk2 was localized to the peripheral adhesive region as well as in the cytoplasm. Very few nucleus stained positive for pPyk2. In 84 advanced serous OC cases, 15 cases (17.9 %) were pPyk2 negative, 37 (44 %) were weakly positive (score 1+), 26 (31 %) were moderately positive (score 2+) and the remaining 6 (7.1 %) were intensively positive (score 3+). Phosphorylated FAK was expressed in 100 % of the high grade serous OC. Among these cases, 5 (6 %) were weakly positive (score 1+), 33 (39.3 %) were moderately positive (score 2+) and 46 (54.7 %) were intensively positive (score 3+). There was no correlation between the intensity of pPyk2 and pFAK expression in OC tissues (*r* = 0.080).

We next examined the correlation of pPyk2 expression with progression-free survival and overall survival. Women with pPyk2 positive tumors had a significantly shorter progression-free survival than women with pPyk2 negative tumors (Fig. 4b). The median progression-free survival of women with pPyk2 positive tumors was 16 months compared to >80 months for women with pPyk2 negative tumors (Log-rank test, *P* = 0.032). We observed a tendency towards decreased overall survival in women with pPyk2 positive tumors with a median overall survival of 46 months compared to 59 months for women with pPyk2 negative tumors (Log-rank test, *P* = 0.087) (Fig. 4b).

### Pyk2 positively regulates ascites-induced ovarian cancer cell migration

**Fig. 5:**
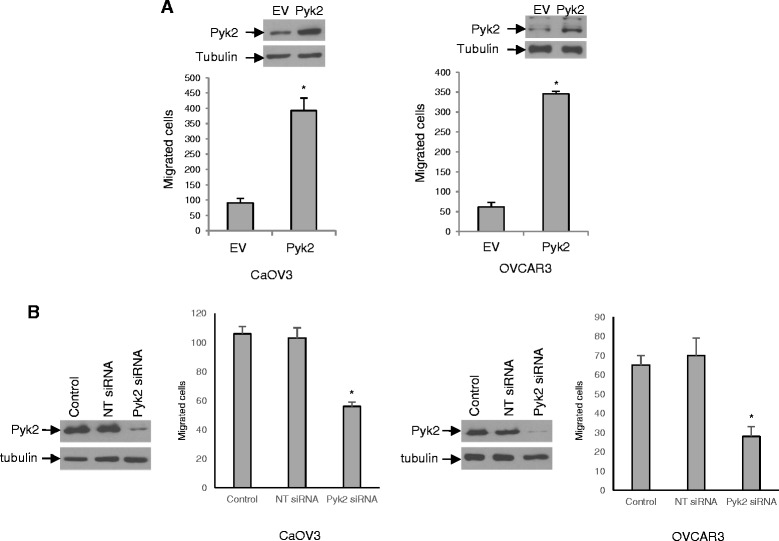
Pyk2 regulates ovarian cancer cell migration. **a** Expression of Pyk2 in CaOV3 and OVCAR3 cells promotes cell migration. Cells were stably transfected to express Pyk2 or empty vector (EV). Immunoblots show the expression of Pyk2 in these cell populations. Cell migration was assessed. **b** CaOV3 and OVCAR3 cells were transfected with Pyk2 siRNA or control non-targeted siRNA (NT siRNA). Pyk2 knockdown was confirmed at 48 h by immunoblot analysis. Cell migration was assessed. Results shown are representative of three independent experiments and data are expressed as the number of total cells that migrated/10 fields. * indicates *P* < 0.001 relative to controls

To examine the role of Pyk2 in OC cell migration, we enforced the overexpression of Pyk2 in OVCAR3 and CaOV3 cells. As shown in Fig. 5a, the expression of Pyk2 was significantly upregulated by the transfection of the Pyk2 vector in OVCAR3 and CaOV3 cells relative to empty vector-transfected cells. Enforced expression of Pyk2 significantly stimulated the migration of OVCAR3 and CaVO3 cells (Fig. 5, *P* < 0.001). Conversely, siRNA-mediated attenuation of Pyk2 protein expression in CaOV3 and OVCAR3 cells significantly decreased cell migration (Fig. 5b, *P* < 0.001). All together, these data suggest that Pyk2 plays an important role in OC cell migration.

### Pyk2 downregulation inhibits CCL18 and ascites-induced cell migration

**Fig. 6:**
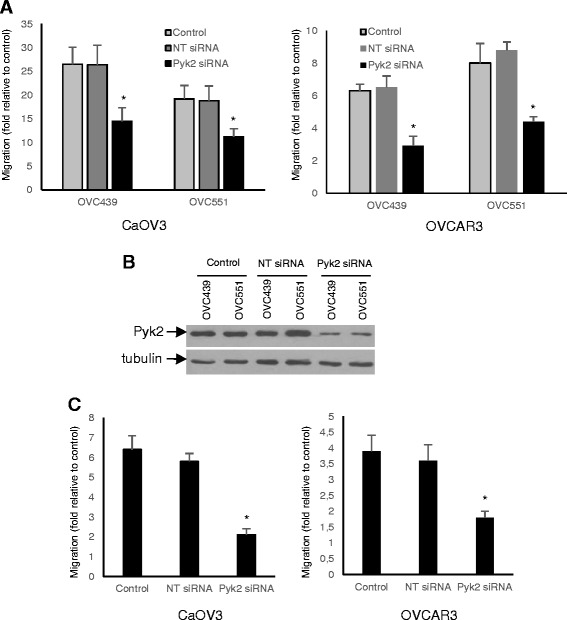
Pyk2 inhibition markedly attenuates ascites and CCL18-induced cell migration. **a** siRNA-mediated downregulation of Pyk2 attenuates ascites-induced migration of CaOV3 and OVCAR3 cells. Cells were transfected with non-targeted (NT siRNA) or Pyk2 siRNA. After 48 h, cells were transferred in Boyden chambers. OC ascites (OVC439 or OVC551) were used as chemoattractants in the lower chambers. Cell migration was measured 24 h later. **b** Immunoblot of CaOV3 cells transfected with NT or Pyk2 siRNA and probed with Pyk2 antibody. **c** CaOV3 and OVCAR3 cells were transfected with non-targeted (NT siRNA) or Pyk2 siRNA. Control corresponds to mock-transfected cells. After 48 h, cells were transferred in Boyden chambers. CCL18 (50 ng/ml) was used as chemoattractants in the low chambers. Cell migration was measured 24 h later. Results shown are representative of three independent experiments and data are expressed as fold increased relative to serum-free incubated cells. *P* values are indicated relative to mock and NT siRNA-transfected cells

To assess the role of CCL18 in ascites in cell migration activity, we examined the effect of downregulating Pyk2 expression. As shown in Fig. 6a, siRNA-mediated downregulation of Pyk2 protein markedly inhibited the migration of CaOV3 and OVCAR3 cells by ascites OVC439 and OVC551 from advanced serous OC patients. Downregulation of Pyk2 protein was confirmed by immunoblot in CaOV3 cell (Fig. 6b).

To confirm the involvement of CCL18 in the induction of OC cell migration, we examined whether the downregulation of Pyk2 could block CCL18-induced migration. As shown in Fig. 6c, the CCL18-induced effect was significantly inhibited by siRNA-mediated attenuation of Pyk2 protein expression in both CaOV3 and OVCAR3 cells. These results suggest that CCL18 in ascites may participate in the induction of migration.

## Discussion

Ovarian cancer is a highly metastatic disease characterized by widespread intraperitoneal dissemination and ascites formation. Cancer-related inflammation plays an important role in OC progression [9]. Chemokine production is associated with chronic inflammation and high levels are found in ascites from advanced OC [6]. Some inflammation-related factors in ascites have been shown to play a pivotal role in pancreatic cancer progression and metastasis [34]. In the present study, we show that CCL18, a C-C chemokine mainly secreted by monocyte-derived cells with M2 phenotype [35], was present at significantly higher levels in ascites obtained from women with advanced serous OC compared to women with benign gynecological conditions. This is consistent with previous data showing high levels of CCL18 in ovarian cancer patients [21, 22]. Although women with high levels of CCL18 had generally a worse outcome compared to women with low CCL18 in our study, the difference did not reach statistical significance. This is perhaps not surprising given the complex nature of OC ascites and the overall outcome is most likely the results of the combined effect of each individual factors affecting tumor cell behavior.

Unlike most carcinomas, OC dissemination is primarily mediated by the shedding of tumor cells from the primary site into peritoneal fluids where they formed free-floating multicellular spheroids that rapidly lead to peritoneal carcinomatosis [36]. Development of pelvic and peritoneal metastasis involves well-defined critical steps, including cell exfoliation, resistance to apoptosis, interaction and adhesion to mesothelial layer, migration and proliferation [36, 37]. We previously demonstrated that OC ascites protect tumor cells from apoptosis through activation of Akt and Erk signaling resulting in the upregulation of anti-apoptotic proteins Mcl-1 and cFLIP [13–15]. This study provide the first evidence that CCL18 in ascites is a factor involved in the stimulation of tumor cell migration. In accordance with this, we found a positive correlation between the levels of CCL18 in ascites and its ability to enhance cell migration, and blocking CCL18 antibodies can inhibit ascites-induced cell migration. The mechanisms of CCL18 action include a direct effect on tumor cells via the activation of Pyk2. This is supported by the following observations. First, siRNA-mediated downregulation of Pyk2 protein expression inhibited the migration response to ascites as well as CCL18-induced migration. Second, the CCL18 levels in ascites were high enough to stimulate the migration of tumor cells and to activate Pyk2. Third, enforced expression of Pyk2 in OC cells markedly enhanced their migration. Finally, siRNA-mediated downregulation of Pyk2 protein significantly attenuated OC cell migration.

In the current study, we demonstrated for the first time that Pyk2 activation in high-grade serous OC was significantly associated with decreased progression-free survival but not with overall survival. In our tissue microarray analysis of 84 clinical samples derived from patients with advanced serous ovarian cancer, women with pPyk2 positive tumors had a 16 months median progression-free survival compared to >80 months for women with pPyk2 negative tumors. This is consistent with previous studies demonstrating an association between Pyk2 expression and worse outcome in hepatocellular carcinomas [38, 39]. Expression of pPyk2 was found in nearly 80 % of advanced serous ovarian cancer patients. It is well established that epithelial-to-mesenchymal (EMT)-associated processes, including cell migration, are determinants for metastasis and therefore poor prognosis. Pyk2 has been reported to induce EMT [40]. Hence, our findings suggest that Pyk2 could enhance EMT and contribute to ovarian cancer progression and metastasis resulting in a worse outcome. Pyk2 integrates ascites signaling to potentiate cell migration. Specifically, we found that Pyk2 is activated by CCL18 in a dose-dependent manner and its depletion leads to substantial inhibition of ascites and CCL18-mediated cell migration. However, CCL18 may not be the only factor in ascites capable of activating Pyk2. Epidermal growht factor (EGF), HER2 and IL-8 receptors have also been shown to activate Pyk2 [41]. These factors have been found to be present in ascites and Pyk2 may therefore integrate signaling from various factors in ascites. This could explain why the levels of CCL18 alone in ascites were not correlated with progression-free survival whereas pPyk2 expression was associated with shorter progression-free survival. Although this would deserve further studies, pPyk2 could represent a new OC prognostic biomarker.

The present study suggests the importance of CCL18 in ascites-mediated cell migration, but this finding does not rule out the possible involvement of other factors from ascites in tumor cell migration. For example, fibronectin, lysophosphatidic acid (LPA), IL-6 and CCL2 have all been shown to enhance OC migration and these factors may be present at high level in ascites [42–45]. The fact that Pyk2 downregulation only partially abrogate ascites-mediated cell migration is in line with this possibiliy and raises the possibility that other factors in ascites can signal through other signaling pathways to stimulate cell migration. It is also possible that CCL18 may affect cell migration through Pyk2-independent signaling pathways. For example CCL18 has been shown to promote metastasis via mTOR and ERK1/2-NF-kB signaling pathways [22, 46].

## Conclusions

In this study, we investigated the contribution of the tumor environment in tumor cell migration. CCL18 was identified as one of the soluble factors responsible for ascites-induced ovarian cancer cell migration through activation of Pyk2. Pyk2 activation was clinically associated with shorter progression-free survival in women with advanced ovarian cancer. Therefore, CCL18 and pPyk2 may represent potential therapeutic targets for OC women.

## Additional file

Additional file 1: Approbation finale du projet de recherche par le Comité d’éthique de la recherche en santé chez l’humain du CHUS (PDF 50 kb)
