# Supplementary material for: CCL18 from ascites promotes ovarian cancer cell migration through proline-rich tyrosine kinase 2 signaling
Source: Mol Cancer. 2016 Sep 9;15(1):58. doi: 10.1186/s12943-016-0542-2 (PMC5017134; doi:10.1186/s12943-016-0542-2)
Supplement: Additional file 1: — Approbation finale du projet de recherche par le Comité d’éthique de la recherche en santé chez l’humain du CHUS (PDF 50 kb) [file 12943_2016_542_MOESM1_ESM.pdf]

Sherbrooke, le 8 décembre 2015

Dr Alain Piche
CHUS
3001, 12e Avenue Nord
Sherbrooke, QC, J1H 5N4

Objet : Approbation finale du projet de recherche par le Comité d'éthique de la recherche en santé chez l'humain du CHUS

Projet #2016-1066 - OVC Ascites
Découverte de biomarqueurs pour le cancer ovarien: exploiter les cytokines présentent dans l'ascite

---

Bonjour Dr Piche,

Le Comité d'éthique de la recherche en santé chez l'humain du CHUS a pris connaissance du **formulaire 20 #5599** déposé dans Nagano le **7 décembre 2015** à la suite de l'approbation conditionnelle du projet cité en rubrique. Les réponses ont été et examinées en comité restreint le **8 décembre 2015.**

Il nous fait plaisir de vous informer que votre projet a été **approuvé de façon finale au CÉR du CHUS le 8 décembre 2015**, et ce, pour une période de 12 mois.(**8 décembre 2016**).

Voici la liste des documents qui sont approuvés:

- Protocole de recherche (Protocol Description of research proposal CRS 2016 - Biomarkers.docx) [date : 2015-10-26, version : 1]
- Ajouter ici tous les documents qui ont besoin d'une approbation éthique. (documents finaux révisés destinés aux patients).

Vous devez attendre l'autorisation du directeur scientifique du Centre de recherche du CHUS avant de débuter la recherche. Certains aspects de la convenance organisationnelle doivent être évalués avant le début du recrutement des participants. Cette autorisation du Centre de recherche s'ajoute à l'approbation du Comité d'éthique.

En acceptant la présente lettre d'approbation finale du Comité d'éthique de la recherche en santé chez l'humain du CHUS, vous vous engagez à soumettre au Comité:

- toute demande de modification au projet de recherche ou à tout document approuvé par le comité pour la réalisation de votre projet.
- tout incidents thérapeutiques graves (SAE) / toutes réactions indésirables graves (SAR) qui sont envoyés au chercheur par le commanditaire sous différentes formes et appellations (IND Safety Letter, Safety Report, Alert Report, etc...) (réf.: CRC-RC5) dans les plus brefs délais.
- tout incidents thérapeutiques graves (SAE) / toutes réactions indésirables graves (SAR) survenant au CHUS **dans les 24 heures** suivant la prise de connaissance par l'équipe de recherche (réf.: CRC-RC6)
- Tout nouveau renseignement sur des éléments susceptibles d'affecter l'intégrité ou l'éthicité du projet de recherche ou d'accroître les risques et les inconvénients des sujets, de nuire au bon déroulement du projet ou d'avoir une incidence sur le désir d'un sujet de recherche de continuer sa participation au projet de recherche;

- Toute modification constatée au chapitre de l’équilibre clinique à la lumière des données recueillies;
- La cessation prématurée du projet de recherche, qu’elle soit temporaire ou permanente;
- Tout problème identifié par un tiers, lors d’une enquête, d’une surveillance ou d’une vérification interne ou externe;
- Toute suspension ou annulation de l’approbation octroyée par un organisme de subvention ou de réglementation;
- Toute procédure en cours de traitement d’une plainte ou d’une allégation de manquement à l’intégrité ou à l’éthique ainsi que des résultats de la procédure.

La présente décision peut être suspendue ou révoquée en cas de non-respect de ces exigences. En plus du suivi administratif d’usage, le CÉR pourra effectuer un suivi actif au besoin selon les modalités qu’il juge appropriées.

En terminant, nous vous rappelons que vous devez conserver pour une période d’au moins un an suivant la fin du projet, un répertoire distinct comprenant les noms, prénoms, coordonnées, date du début et de fin de la participation de chaque sujet de recherche.

Il est à noter qu'aucun membre du comité d'éthique participant à l'évaluation et à l'approbation de ce projet n'est impliqué dans celui-ci.

En ce qui concerne l'essai clinique visé, à titre de représentant du Comité d'éthique de la recherche, je certifie que:

1. La composition de ce Comité d'éthique satisfait aux exigences pertinentes prévues dans le titre 5 de la partie C du Règlement sur les aliments et drogues.
2. Le Comité d'éthique de la recherche exerce ses activités de manière conforme aux bonnes pratiques cliniques, et
3. Ce Comité d'éthique a examiné et approuvé le formulaire de consentement et le protocole d'essai clinique qui sera mené par le chercheur susmentionné, au lieu d'essai indiqué. L'approbation et les opinions du présent comité ont été consignées par écrit.
4. Ce Comité est conforme aux normes américaines. (FWA #00005894)

Je vous prie d’accepter, Dr Piche, mes meilleures salutations.

Annabelle Cumyn, MDCM, MHPE
Présidente du comité d'éthique de la recherche chez l'humain
